# Overcoming treatment resistance in acute promyelocytic leukemia and beyond

**DOI:** 10.18632/oncotarget.1244

**Published:** 2013-08-04

**Authors:** Tsz Kan Fung, Chi Wai Eric So

**Affiliations:** Leukaemia and Stem Cell Biology Group, Department of Haematological Medicine, King's College London, London SE5 9NU, UK; Leukaemia and Stem Cell Biology Group, Department of Haematological Medicine, King's College London, London SE5 9NU, UK

From the introduction of *all-trans* retinoic acid (ATRA) to the recent development of arsenic trioxide (ATO) treatment, acute promyelocytic leukemia (APL) characterized by the presence of retinoic acid receptor alpha (RARA) fusion has been transformed from a highly fatal cancer to a highly curable disease. In spite of this unprecedented success, there are still a significant number of high-risk patients who fail to achieve a complete molecular remission or relapse and become resistant to the treatments. Here we discuss the underlying mechanisms and the potential avenue of targeting a critical histone demethylase PHF8 in overcoming the treatment resistance in APL and beyond.

**Figure 1:**
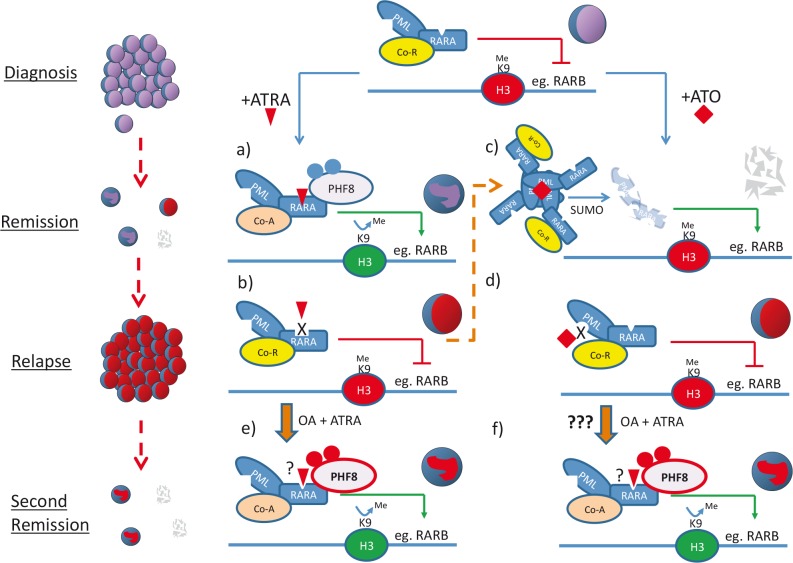
Schematic diagram illustrates the molecular basis of ATRA/ATO treatments and the potential avenues of overcoming treatment resistance by modulating PHF8 activity in APL PML-RARA aberrantly recruits co-repressor complexes (Co-R, yellow oval) and epigenetically suppresses expression of downstream target genes critical for differentiation and tumor suppression. (a) ATRA (red triangle) binds to the LBD of RARA moiety and facilitates the exchange of co-repressor with co-activator complexes (co-A, pink oval). ATRA also induces phosphorylation of PHF8 that is recruited by the RARA fusion to remove suppressive H3K9me epigenetic mark and subsequently converts repressive chromatin (red H3 oval) to permissive chromatin (green H3 oval) for active transcriptional programs leading to cell differentiation. (b) However, long-term exposure to ATRA may cause selection or induction of leukemic clones carrying mutations on the LBD of the RARA moiety or aberrant transcription repression complexes that could not be dissociated by pharmacological level of ATRA treatment. Hence cells become ATRA-resistant and APL relapses. (c) On the other hand, ATO (red diamond) that leads to degradation of PML-RARA has been shown effective in inducing complete remissions even for ATRA-resistant APL (thin dashed orange arrow). In contrast to ATRA, a high dose of ATO results in apoptosis of APL cells. (d) Again, long-term treatment of ATO can select/induce resistant leukemic clone(s) carrying mutations on the PML moiety affecting ATO-mediated degradation. ATO-resistant clone expands and disease relapses. (e) and (f) Over-expression or hyperphosphorylation of PHF8 (red), which could be achieved by phosphatase inhibitor OA treatment, re-sensitizes ATRA-resistant APL to ATRA, probably through epigenetic activation of downstream target gene expression (e). Since ATO-resistance in APL is largely due to the mutation on PML moiety, PHF8 can still in principle be effective in targeting the RARA moiety of the fusion proteins and induces cell differentiation (f).

As a result of international collaborative research efforts, APL has led the way in demonstrating the promises and defining important principles of successful targeted therapies. At the same time, it also reveals various challenges that targeted therapy will face. Among them is the drug resistance that has taken the central stage. Although the highly specific and effective way of targeting the critical oncogenic events that drive the diseases represents a major advantage over the generic highly toxic chemotherapy, this is a double-edged sword that also makes targeted therapy particularly susceptible to treatment resistance. In APL, ATRA specifically binds to and alters the conformation of oncogenic RARA fusion leading to de-repression of downstream targets by epigenetic reprogramming and subsequent degradation of the fusion proteins, leading to differentiation of APL blasts (Figure 1a). However, a prolonged ATRA treatment can result in drug resistance by inducing and/or selecting leukemic clones carrying mutations on the ligand binding domain (LBD) of the RARA moiety or aberrant transcription repression complexes that could not be dissociated by ATRA treatment (Figure 1b), which are commonly found in relapse cases. Similar problems also occur in the ATO treatment. PML-RARA fusion accounts for over 98% of APL. Mechanistically, ATO binds directly to PML moiety of the PML-RARA fusion that induces direct cross linking, SUMOylation and subsequent degradation of the fusion protein. In contrast to ATRA, a high dose of ATO triggers apoptosis of APL cells without a significant induction of differentiation (Figure 1c). Although ATO has been successfully used both in combination with ATRA for induction therapy and as a second line treatment for ATRA-resistance, mutations on the PML moiety of the RARA fusion affecting the ATO-induced protein degradation can be found in APL cells after ATO treatment (Figure 1d). Moreover APL patients carrying double mutations affecting both ATRA binding and ATO-mediated degradation have also been reported, indicating that a better therapeutic strategy is urgently needed to prevent and/or overcome the treatment resistance.

While targeting oncogenic transcription factors with small molecule inhibitors has been proved difficult, their associated epigenetic modifying enzymes such as histone deacetylase and DNA methyltransferases with rigid catalytic domains have been revealed as potential therapeutic targets in various cancers. To search for critical factors involved in ATRA response, our lab has discovered histone demethylase PHF8 as a key mediator that governs the ATRA sensitivity in APL cells, and assigns a new function of this class of epigenetic modifying enzymes in mediating treatment response/resistance (Arteaga et al., Cancer Cell, 2013, 23: 376-89). PHF8 is recruited to PML-RARA upon ATRA treatment to remove the repressive transcriptional mark but enhances activation mark of PML-RARA downstream target genes. Over-expression or enhanced phosphorylation of PHF8 induces epigenetic reprogramming that resurrects both in vitro and in vivo ATRA-sensitivity to resistant APL cells carrying the LBD mutant of PML-RARA or forming aberrant transcriptional repressor complexes associated with the fusion (Figure 1e). In addition, PHF8 can also overcome ATRA-resistant associated with the variant APL fusion, PLZF-RARA that cannot be targeted by ATO. As a proof of principle, pharmacological inhibition of PHF8 dephosphorylation by okadaic acid (OA) re-sensitizes ATRA-resistant APL cells to the treatment and significantly extends the disease latency in animal models, suggesting PHF8 activator may help to overcome ATRA-resistance. Interestingly, since PHF8 specially binds to region C of the RARA moiety to mediate transcriptional activation and subsequent degradation of the fusion, it is tempting to speculate that PHF8 may be useful in tackling ATO-resistant APL cells carrying mutations on the PML moiety (Figure 1f). On other other hand, identification of PHF8 as a major mediator for ATRA response can also have important implications to the potential application of ATRA to other cancers. While the RARA fusion is the major target of PHF8-mediated ATRA response in APL cells, wild type RARA may also interact with PHF8 to mediate ATRA response in other cell types. It is evidenced that over-expression of PHF8 can facilitate neuronal differentiation of embryonic carcinoma cells. It will be of interest to assess if PHF8 may also act as a positive retinoic acid modulator (RAM) and manipulation of its activity can confer ATRA sensitivity to other cancer subtypes, in particular neuroblastoma where retinoic acid signaling has already been implicated.

With the improved understanding of the molecular basis of cancer, an increasing number of target therapies have been developed and some already show promises in early phases of various clinical trials. While these therapies will very likely improve the complete remission rate for certain cancers and minimizes the highly toxic side effects associated with chemotherapy, it is foreseeable that drug resistance will be observed in a significant number of patients undergoing these highly specific treatments. Tackling treatment resistance will likely be a major challenge of any future targeted therapy. Having better understanding the mechanisms underlying the treatment response will provide unprecedented opportunities to predict and overcome treatment resistance in the refractory subset of patients. APL can be not only an inspiring story in cancer research but also a prototype for translating the success to other cancers.

